# The Role of Autopsy and Post-Mortem Investigations in Falling Traumas in the Hospital Environment

**DOI:** 10.3390/diagnostics12123168

**Published:** 2022-12-14

**Authors:** Matteo Antonio Sacco, Fabrizio Cordasco, Ludovico Abenavoli, Angelo Lavano, Giovanni Gallotta, Eugenio Garofalo, Andrea Bruni, Carmen Scalise, Saverio Gualtieri, Alessandro Pasquale Tarallo, Valerio Riccardo Aquila, Pietrantonio Ricci, Isabella Aquila

**Affiliations:** 1 Institute of Legal Medicine, Department of Medical and Surgical Sciences, “Magna Graecia” University, 88100 Catanzaro, Italy; 2 Department of Health Sciences, “Magna Graecia” University, 88100 Catanzaro, Italy; 3 Unit of Neurosurgery, Department of Medical and Surgical Sciences, “Magna Graecia” University, 88100 Catanzaro, Italy; 4 Department of Clinical and Experimental Medicine, Federico II University, 80138 Naples, Italy; 5 Anesthesia and Intensive Care Unit, Department of Medical and Surgical Sciences, “Magna Graecia” University, 88100 Catanzaro, Italy; 6 Department of Medical and Surgical Sciences, “Magna Graecia” University, 88100 Catanzaro, Italy

**Keywords:** forensic pathology, falls, trauma, hospital, autopsy

## Abstract

Falls in a hospital setting are a global public health problem. Despite the production of sensors and various preventive tools to reduce the risk, falls remain a dangerous event with a significant impact on the morbidity and mortality of patients. Despite numerous prevention strategies, falling in the hospital are not always investigated and the autopsy is not always performed in these cases, so it is often not known whether the death is related to the fall or to other causes, inevitably affecting the assessment of any profiles of medical liability for health personnel or for the hospital in the accident. We describe three cases of falls that occurred in different hospitals, in which the autopsy allowed to diagnose with certainty the extent of the trauma and to reconstruct its dynamics. Along with the circumstantial and documentary analyses, deficiencies both in the trauma diagnostics and in the application of the safety measures on the patients were showed. Together with the description of our cases, we propose the post-mortem investigations of these events, both for judicial and risk management purposes.

## 1. Introduction

The fall is defined as a sudden downward movement of a subject with knocking over to the ground. Falls represent a relevant public health problem and are the most common adverse event in the hospital setting with fatal consequences for patient health [1]. According to the WHO, falls are the second leading cause of accidental death in the world with an estimated 650,000 deaths/year globally [2]. In the USA, the fall rate ranges from 1.7 to 25 falls per 1000 patient-years, mainly affecting geriatric and psychiatric patients. The largest proportion of patients involved are over 65 years old (30%) or over 80 years old (50%) [2,3,4]. The impact on the victims’ health is considerable with both physical and psychic effects. The physical effects mainly concern the risk of fractures with related complications but also damages deriving from the involvement of vital organs or multiple anatomical sites [5,6,7]. Half of the elderly who report a hip fracture can no longer walk and 20% of them develop complications that lead to death within six months [8]. The psychic effects concern the risk of entrapment and long-term hospitalization of the subject after the trauma with related repercussions on the mental health state and on social relationships [9]. The most frequent victims are therefore frail subjects, i.e., elderly people or patients with the impossibility or difficulty of walking or subjects presenting dementia, cognitive decline, mental retardation or psychiatric diseases [10]. Therefore, the fall significantly affects the patient’s quality of life with relevant effects on disability, morbidity and finally on mortality. A significant proportion of falls are predictable events and therefore also preventable. According to the Recommendation n. 13 of November 2011 published by the Ministry of Health in Italy, only 8% of falls that occur in hospital can be considered unforeseeable or random events [8]. The remaining portion of the events is divided into accidental events (14%), due to environmental factors (e.g., slippery floor), or they can be events in patients with already known risk factors (e.g., subjects with walking difficulties or disoriented patients) (78%) [8]. Therefore, in most cases, falls in hospital settings are preventable and it is therefore possible to identify profiles of liability for operators involved or the healthcare facility. In this regard, despite numerous papers about fall risk prevention and patient risk factors, little is known in the literature about the post-mortem diagnosis of fall trauma and about the forensic implications of these cases.

When an accidental fall occurs, it is essential that diagnostic and management protocols are promptly initiated, with adequate timing and instrumental investigations, as the risk of mortality is very high [11]. Furthermore, in many cases, the falling trauma may not cause immediate clinical effects or cause subtle signs with delay in the application of diagnostic protocols; other times the event may remain unacknowledged by family members and the Judicial Authorities during hospitalization until death. Moreover, since the victims are frail patients, often suffering from psychiatric or neurological pathologies such as to make communication more difficult, the clinical diagnosis and reporting of the event are complex [12,13]. Further, there is difficulty in sorting out natural from accidental falls—distinguishing falls from syncope, weak balance, and coordination, and slips and other true accidents. For this reason, it is crucial to understand how the trauma occurred and whether the event could have been prevented. Unfortunately, in many of these cases the autopsy is not performed so it is not always possible to determine with certainty the cause of death and the actual extent of the trauma. This limit certainly affects the assessment of the dynamics of the event, both for judicial and risk management purposes. The aim of this work is to analyze the role that the proper interpretation of autopsy data and forensic investigations play in the post-mortem diagnosis of fall trauma in hospital settings. We describe three cases reported from our experience, in which the autopsy data collected have assumed importance not only in determining death but above all in the evaluation of deficiencies related to poor safety and vigilance of in-patients.

## 2. Materials and Methods

The authors have collected various cases relating to falls occurred in a hospital environment (2009–2022) and the collection is in progress through a prospective method, through the help of a multidisciplinary team made up of forensic pathologists, anesthesiologists, internists, geriatricians, and neurosurgeons. Three peculiar cases have been selected in which the investigators carried out autopsies and examined health records. Forensic investigators performed photographic surveys of the scene and injuries. The external examination was carried out with detailed analysis and measurement of the injuries. The lesions were also incised to determine their vitality. An internal examination was carried out with evaluation of all organs. During the autopsy, samples of biological fluids (blood, urine, vitreous humor) were taken. The fluids were analyzed first by qualitative immunofluorescence method and then by automated immunoassay on an ILAB 600 Chemistry Analyzer (Instrumentation Laboratory, Bedford, MA, USA). Subsequently, the tissue samples taken were stored in formalin and then the microscopic histopathological examination was performed.

In all cases, the collection of clinical data was carried out through the study of the medical records and all the instrumental tests carried out during hospitalization. The possible presence of intrinsic or extrinsic risk factors was investigated. Particular attention was paid to the analysis of difficulty walking; study of psychiatric comorbidities; pharmacological treatment (analysis of antihypertensive, hypoglycemic, psychiatric drugs); vision or hearing impairments. As part of the extrinsic factors, environmental data were evaluated (flooring, lighting, presence of stairs, height of beds, and application of safety measures or any means of restraint).

It was then investigated how the fall had occurred and whether the patient was bedridden or in a wheelchair at the time of the event. The autopsy, documentary and circumstantial data were then compared to determine a probable dynamic. Finally, the safety measures were examined, and the clinical and instrumental diagnostic protocol performed at the time of the event was evaluated [14].

## 3. Cases Presentation

### 3.1. Case 1

**Figure 1:**
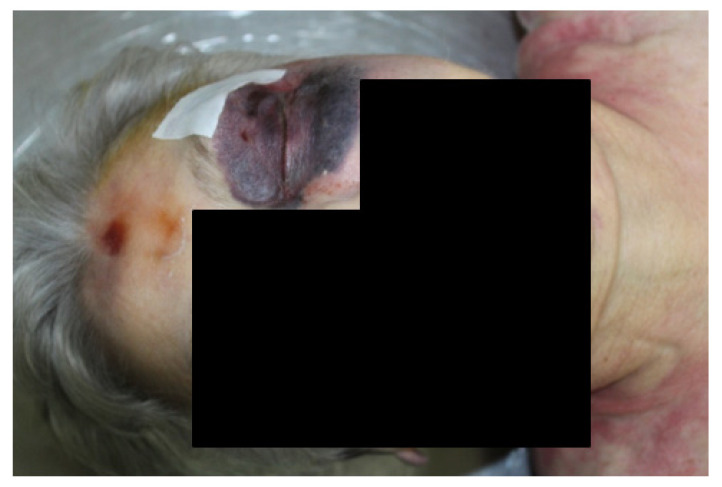
Facial trauma in case 1.

An 82-year-old patient died in a nursing home. The patient had a history of paranoid schizophrenia with severe cognitive impairment, incontinence, COPD, fracture of the right femur. From the clinical diary it emerged that the patient was found dead under sudden circumstances. The clinical diary described that the day before her death the patient had accidentally fallen and reported a lacerated wound on the left brow region which had been stitched up. At the time of the necropsy visit, before closing the coffin, however, the physician noted a very conspicuous purplish ecchymosis overall periorbital and left zygomatic region as well as a bruise on the frontal region (Figure 1). In doubt of a probable traumatic cause of death, he informed the Judicial Authority which ordered the autopsy.

### 3.2. Case 2

A 58-year-old patient was in a COVID-19 hospital ward. From the collection of data from a witness, the patient was on the stretcher during the transport procedures, in a probable attempt by the physicians to lower the height of the stretcher, it suddenly lowered, falling to the ground for a height of about one meter, causing a head injury. The patient appeared on that day apparently stable from a neurological point of view. The following day, due to the worsening of the clinical conditions, the patient was transferred following a CT examination to the neurosurgery unit to undergo decompression surgery for cerebral hemorrhage. The patient remained in a coma and died two days later, following the observation of brain death.

### 3.3. Case 3

An 80-year-old patient suffering from ischemic stroke with left hemiplegia, senile dementia, anxious-depressive syndrome, osteoporosis, previous femur fracture, was hospitalized in a nursing home. The clinical diary described an accidental fall due to slipping from the wheelchair used for transfers of the patient as she was not self-sufficient in walking. Following radiological evaluation (brain CT) the doctors requested neurosurgical advice with transfer by 118 with helicopter rescue. Information was subsequently given by the Neurosurgery unit where the patient had been transferred of a worsening of the health conditions of the patient with death.

## 4. Results

### 4.1. Analysis of the Collected Cases

The analysis of the cases evaluated over 50 episodes of falls examined both in the criminal and civil judicial fields for compensation purposes. Table 1 shows in detail the surveys carried out, the results and the predictable and unpredictable risk factors highlighted.

### 4.2. Autopsy and Forensic Findings

#### 4.2.1. Case 1

**Figure 2:**
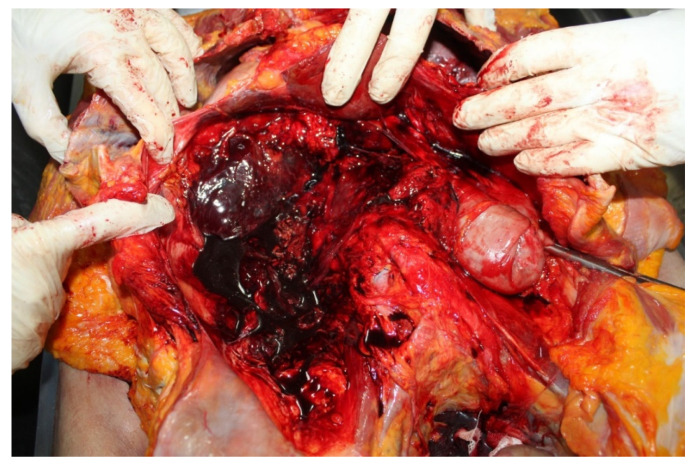
Abdominal trauma at autopsy in case 1.

**Figure 3:**
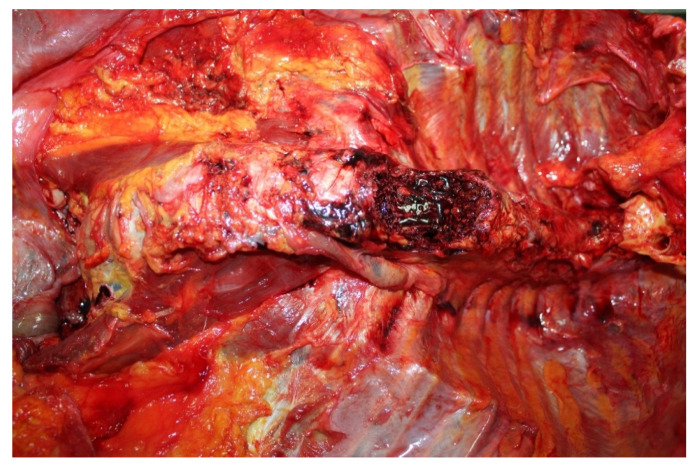
Vertebral fractures (T11-T12) at autopsy in case 1.

The external examination revealed large bruises of an intense purplish color over the entire left periorbital region measuring 8 cm × 8 cm, extending up to the zygomatic region with swelling of the tissues. There was also left conjunctival hemorrhage; bruise on the frontal region with dimensions of 4 cm × 4 cm. Internal examination showed cranial and thoracoabdominal polytrauma with subarachnoid hemorrhage, multiple rib fractures, vertebral fractures, hepatic hemorrhage (Figure 2 and Figure 3). The toxicology test gave negative results. The examination of the clinical records showed that the patient, following the fall, with unclear circumstances, did not perform diagnostic instrumental tests. The autopsy showed objective findings of death occurred from polytrauma involving the cranial, thoracic, and abdominal regions.

#### 4.2.2. Case 2

**Figure 4:**
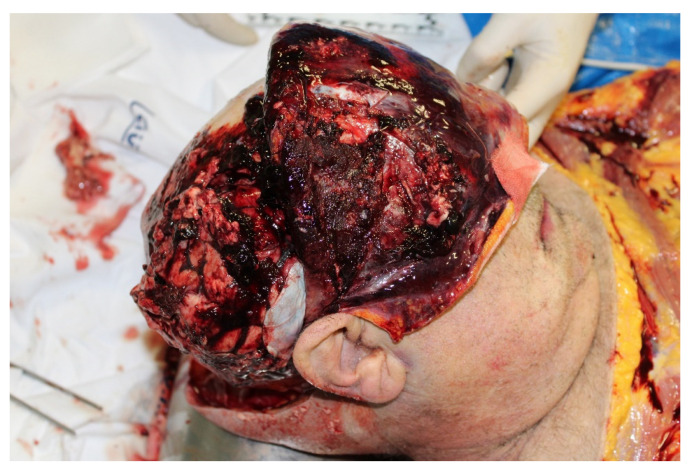
Cerebral hemorrhage in case 2.

The external examination showed results of surgery with decompression on the right temporal region as well as extensive hematoma on the right hip and right leg. At the autopsy, hemorrhagic infiltration of the soft head tissues as well as of the skull was noted with massive cerebral hemorrhage and loss of its regular structure (Figure 4). The records examination showed that on the day of the fall, the patient was apparently lucid so that the doctors did not deem it necessary to carry out radiological tests. The death, therefore, occurred two days after the fall, following surgery, due to head trauma with cerebral hemorrhage.

#### 4.2.3. Case 3

Forensic investigations showed that the patient had a cerebral hemorrhage with fatal neurological complications such as to cause death the day after the fall, despite transfer to the neurosurgical unit. From circumstantial data and from the photographic investigations carried out by the Judicial Police, it was discovered that the wheelchair from which the patient fell appeared with the support base inclined upwards and with the back inclined backwards and that it did not was safe. The dynamics reconstruction showed that the patient, in the absence of a safety belt, having no control of the functions of her head and torso, accidentally fell forward hitting her head on the floor. The records examination showed that the patient had already fallen from the wheelchair a month earlier, suffering a fracture of the nasal septum with mild head trauma. Also in this case, no radiological examinations were carried out at the time of the event.

## 5. Discussion

Falls represent 38% of adverse events in the hospital setting, constituting a relevant event [15]. A percentage between 4 and 6% of falls causes major injuries, i.e., bleeding, fractures, and subdural hemorrhage [16]. The compression of the patient’s fall risk in a healthcare facility is an indicator of the quality of the healthcare service and cannot be achieved without specific training of the healthcare personnel to improve the competence in the prevention and management of falls, but also the awareness of the risk [17]. The scientific literature has described various criteria for identifying patients at risk and for establishing a real therapeutic education path that primarily involves healthcare professionals. For this purpose, various assessment scales have been identified such as patient autonomy scales, scales aimed at framing the risk of falling and mobility, environmental or multifactorial assessment scales. Fernández-Bermejo Ruiz et al. have described a monitoring system of the patient’s position in bed with sensors to avoid dangerous situations [18]. Cortes et al. contested the real effectiveness of these systems on support surfaces such as beds or chairs, suggesting the use of alternative wearable sensors [19]. Despite the study of various prevention systems, unfortunately falls in hospitals remain a very frequent adverse event with relevant forensic implications [20].

**Figure 5:**
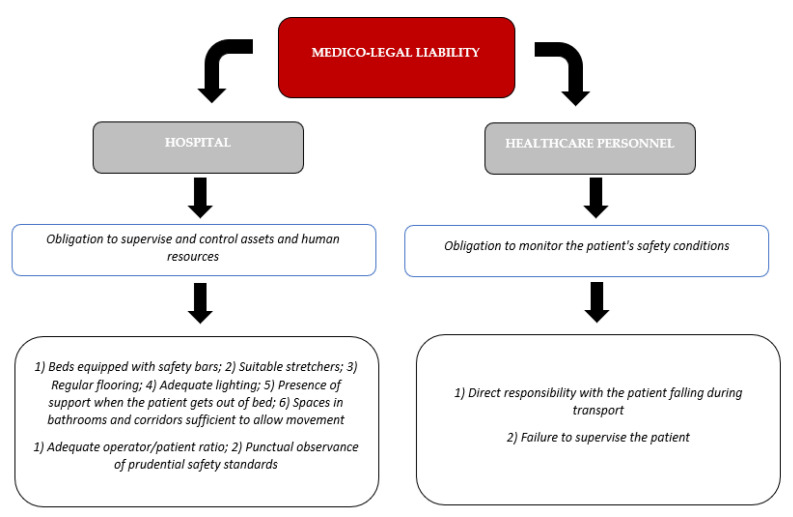
Profiles of liability due to falling in hospital environment.

Liability related to accidental falls in a nosocomial environment may involve different spheres of competence, including a liability of the hospital directorate or a responsibility of the healthcare personnel. The responsibility of the hospital directorate is identified as the owner of the asset and therefore adequate supervision and control is required. It regards the safety of environments and facilities, namely: (1) Beds equipped with protective bars; (2) Suitable stretchers; (3) Regular flooring; (4) Adequate lighting; (5) Presence of support or when the patient gets out of bed; (6) Spaces in bathrooms and corridors sufficient to allow movement. Furthermore, the control with suitable organization of human and structural resources is expected through (1) Adequate operator/patient ratio; (2) Punctual observance of prudential safety standards (Figure 5).

The responsibility of the healthcare personnel is identified as the operators are bearers of a position of guarantee towards the patients and have the obligation to protect their health. The responsibility of healthcare personnel involves all professionals, i.e., physicians, nurses and health care assistants, and may be due to: (1) Direct responsibility with the patient falling during transport (e.g., falling during transport on a stretcher; falling during transfer from bed to bed; fall in ambulance during transfer to hospital); (2) Failure to supervise the patient (Fall of non-self-sufficient patients not supervised; failure to assess the patient’s risk of falling; failure to apply fall prevention measures (e.g., bed rails and wheelchair seat belt).

**Figure 6:**
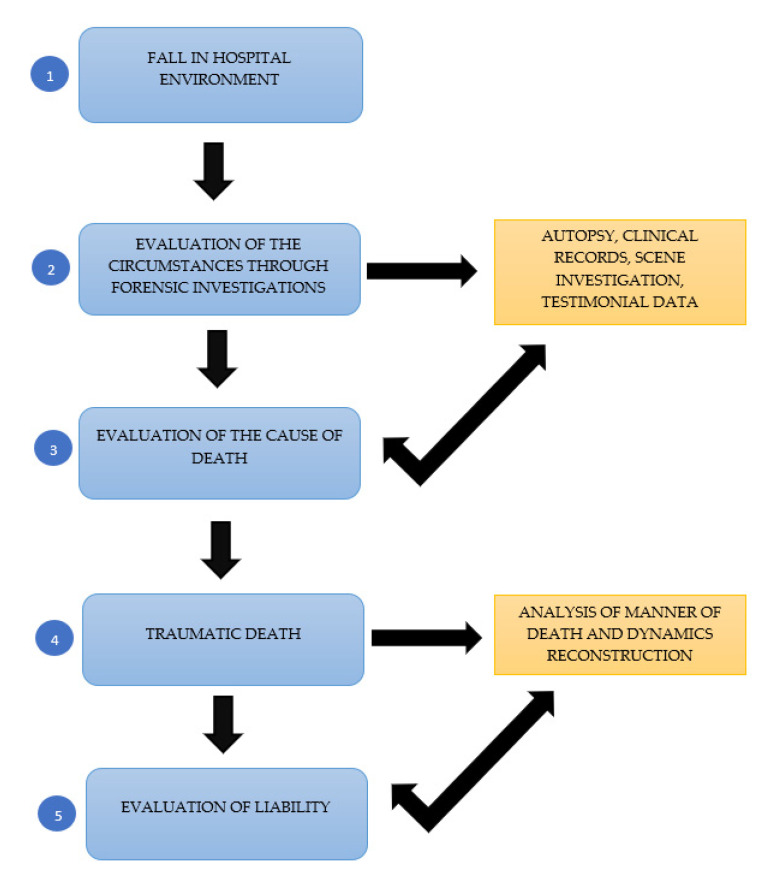
Post-mortem diagnostic protocol in case of trauma.

The role of healthcare professionals is crucial in the management of these events [21]. When an accidental event occurs, it is essential that operators activate diagnostic protocols immediately. This implies both a timely physical and neurological examination (with examination of the Glasgow coma scale, analysis of any fractures and/or traumas, estimation of the severity of the trauma) by virtue of the anatomical site involved but also the performing of appropriate and timely radiological investigations. The patient also requires careful monitoring of the signs and symptoms and the possible serial repetition of the radiological investigations [22]. We recommend the assessment of the trauma and the analysis of therapeutic decisions by a multidisciplinary team that always includes specialists (radiologist, orthopedist, geriatrician, psychiatrist). Furthermore, in the clinical diary we highlight the importance of reporting the fall event, detailing the manner of the event in order to allow for a precise reconstruction. Where the trauma compromises the patient’s health or in cases of death that occurred within a short time from a trauma or doubtful cases in which there is even just a suspicion of association of death with a trauma, the Judicial Authority must be promptly informed. For this reason, autopsy should be mandatory especially when it is not clear why someone fell (natural or accident). Post-mortem diagnostics should therefore always include forensic investigations aimed at ascertaining the manner of the fact but also the analysis of the medical records, and the judicial inspection [23]. In particular, the autopsy represents the gold standard for post-mortem diagnosis of traumas as it allows to collect objective findings about the cause of death. Therefore, the careful interpretation of objective data collected by forensic pathologist (autopsy, laboratory, clinical, imaging data) and further investigations, play a decisive role in the analysis of the event. This, in turn, allows us to understand the quality of the healthcare service offered to the patient, evaluating the correct application of the surveillance and safety measures during hospitalization [24]. In all three cases described, the autopsy allowed to collect data about the traumatic nature of the death and to start the investigations (Figure 6).

In the cases reported, the patients were not self-sufficient as they suffered from pathologies that did not allow them to walk and therefore required close supervision. In case 1, there was an omission of vigilance during mobilization as the patient, suffering from dementia, accidentally fell, causing a serious trauma which was not reported by the physicians. Forensic data collected by testimonials showed that the patient fell while trying to get up independently from a chair and walk, in the absence of a seat belt. In the diagnostic field, moreover, the patient did not perform the appropriate radiological investigations and only the intervention of the physician at the moment of the necroscopic evaluations allowed the post-mortem investigations to be started, in the absence of which the case would never have come to the attention of the Judicial Authority. In this case the death certificate was completely amended because the trauma has not been reported. In case 2, there was a liability of the healthcare personnel for failure to supervise the safety of the means of transport; also, in this case the diagnosis of trauma had not been timely as radiological investigations were omitted. In this case, autopsy allowed to confirm that head trauma was the primary cause of death because the patient suffered also from a severe bilateral COVID-19 pneumonia with respiratory and renal insufficiency. In case 3, an omission was discovered in the application of the safety belt on the wheelchair; also, in this case the correct protocols for the diagnosis had not been started, and the patient was transferred to a neurosurgical unit with advanced cerebral hemorrhage. Further, autopsy allowed to confirm that the trauma was the main cause of death because the patient had also multiple systemic comorbidities.

Therefore, autopsy showed objective findings which were described in the autopsy report. The reliable body of evidence given by autopsy and further investigations led to helped to bring it to light all the following points:

− An omission in the patients supervision;
− An inappropriate diagnosis of the trauma at the moment of the fact;
− An incorrect reporting of the event in diary;
− Poor prevention with lack of anti-fall safety devices;
− A delay in the therapeutic treatment of patients.

The limitation of this study is related to a still numerically limited case series. We look forward to the collection of other cases of falls in hospital settings and above all the comparison with other forensic institutes in order to evaluate uniform post-mortem protocols for the management of these cases and the related effects for falling risk prevention.

## 6. Conclusions

In conclusion, despite the spread of multiple risk prevention systems, falls remain very dangerous events. We emphasize the correct analysis in cases of deaths from accidental falls in a hospital setting. A careful collection of objective forensic data including autopsy findings and all forensic investigations described are necessary, in view of the case history, to formulate a proper interpretation of the event. Investigation of a fall might reveal information that could be useful in preventing others from a similar fall.

## Figures and Tables

**Table 1:** Results about the collected cases of falls.

Place	Years	Number of Cases	Forensic Investigations	Results	Predictable Factors	Non Predictable Factors
Institute of Legal Medicine-Magna Graecia University of Catanzaro	2009-ongoing	>50	-Autopsy -Toxicological examination; -Histopathological examination; -Scene analysis; -Evaluation of testimonials	-Evidence of preventable risk factors (80%); -Structural deficiencies (12%); -Unpredictable factors (8%)	-Psychiatric pathologies; -Dementias; -Walking difficulties (results of fractures, advanced age, obesity, etc.)	-Accidental falls not related to environmental deficiencies (syncope/sudden loss of balance); -Voluntary attempts of unauthorized autonomous walking

## Data Availability

Not applicable to this article as no datasets were generated.
